# Conventional Therapy and Promising Plant-Derived Compounds Against Trypanosomatid Parasites

**DOI:** 10.3389/fmicb.2012.00283

**Published:** 2012-08-06

**Authors:** Daniela Sales Alviano, Anna Léa Silva Barreto, Felipe de Almeida Dias, Igor de Almeida Rodrigues, Maria do Socorro dos Santos Rosa, Celuta Sales Alviano, Rosangela Maria de Araújo Soares

**Affiliations:** ^1^Laboratório de Estruturas de Superfície de Microrganismos, Instituto de Microbiologia Prof. Paulo de Góes, Universidade Federal do Rio de JaneiroRio de Janeiro, Rio de Janeiro, Brazil; ^2^Laboratório de Biologia de Protistas, Instituto de Microbiologia Prof. Paulo de Góes, Universidade Federal do Rio de JaneiroRio de Janeiro, Rio de Janeiro, Brazil; ^3^Laboratório de Quimioterapia Experimental para Leishmaniose, Departamento de Microbiologia Geral, Instituto de Microbiologia Prof. Paulo de Góes, Centro de Ciências da Saúde, Universidade Federal do Rio de JaneiroRio de Janeiro, Rio de Janeiro, Brazil; ^4^Instituto de Química Programa de Pós Graduação em Ciência de Alimentos, Centro de Ciências Matemáticas e da Natureza, Universidade Federal do Rio de JaneiroRio de Janeiro, Rio de Janeiro, Brazil

**Keywords:** leishmaniasis, Chagas disease, sleeping sickness, *Trypanosoma* spp., *Leishmania* spp., chemotherapy, medicinal plants, phytotherapy

## Abstract

Leishmaniasis and trypanosomiasis are two neglected and potentially lethal diseases that affect mostly the poor and marginal populations of developing countries around the world and consequently have an important impact on public health. Clinical manifestations such as cutaneous, mucocutaneous, and visceral disorders are the most frequent forms of leishmaniasis, a group of diseases caused by several *Leishmania* spp. American trypanosomiasis, or Chagas disease, is caused by *Trypanosoma cruzi*, a parasite that causes progressive damage to different organs, particularly the heart, esophagus, and lower intestine. African trypanosomiasis, or sleeping sickness, is caused by *Trypanosoma brucei* and is characterized by first presenting as an acute form that affects blood clotting and then becoming a chronic meningoencephalitis. The limited number, low efficacy, and side effects of conventional anti-leishmania and anti-trypanosomal drugs and the resistance developed by parasites are the major factors responsible for the growth in mortality rates. Recent research focused on plants has shown an ingenious way to obtain a solid and potentially rich source of drug candidates against various infectious diseases. Bioactive phytocompounds present in the crude extracts and essential oils of medicinal plants are components of an important strategy linked to the discovery of new medicines. These compounds have proven to be a good source of therapeutic agents for the treatment of leishmaniasis and trypanosomiasis. This work highlights some chemotherapeutic agents while emphasizing the importance of plants as a source of new and powerful drugs against these widespread diseases.

## Introduction

Leishmaniasis and trypanosomiasis are neglected tropical diseases caused by protozoans of the Trypanosomatid family, a diverse group of flagellated parasites that show similar cellular structures and undergo morphological alterations during their life cycles. The human diseases caused by trypanosomatids such as leishmaniasis, African trypanosomiasis (or sleeping sickness), and American trypanosomiasis (or Chagas disease) are transmitted by insects that affect 20 million people and cause 100,000 deaths per year, primarily in the tropical and subtropical areas of the world. In these regions, half of a billion people are at risk of infection (Stuart et al., 2008).

According to the World Health Organization (WHO), an ideal drug for the treatment of parasitic diseases should fulfill the following requirements: (i) parasitological cure in all the phases of the disease; (ii) effective in single or few doses; (iii) low cost for the patients; (iv) no collateral or teratogenic effects; (v) no need for hospitalization; and (vi) no induction of resistance. As described below, this ideal drug does not exist for the treatment of these parasitoses, and it will take a long time before such a drug is available. Additionally, because the vaccine approach has not produced satisfactory results in clinical trials, chemotherapy based on drugs that are not highly effective and cause side effects is the only treatment available for these maladies.

Considering the low number and efficacy of drugs available for the treatment of these diseases as well as their side effects and the resistance developed by parasites, the research in phytosciences, mainly regarding the properties of bioactive phytocompounds in the crude extracts and essential oils of medicinal plants, may lead to the discovery of new medicines with appropriate efficiency that are more accessible to the patients (Alviano and Alviano, 2009). Overall this review aims to provide a better understanding of the recent developments of the phytosciences to treat leishmaniasis and trypanosomiasis neglected tropical diseases in terms of drug discovery and development in the world today.

## Leishmaniasis, Chagas Disease, and Sleeping Sickness

Leishmaniasis is a generic term for diverse clinical manifestations including cutaneous, mucocutaneous, and visceral disorders caused by species of the genus *Leishmania*. All *Leishmania* species display similar morphologies and present two main developmental stages throughout their life cycles. The extracellular replicative forms, the promastigotes, are found in the gut of the insect vectors, the phlebotomines sandfly. The amastigote is the obligatory intracellular form observed in the mononuclear phagocytic system cells of vertebrate hosts. During blood feeding, the infected sandflies expel, together with saliva, a number of infective promastigotes found at the cardiac valve. Cutaneous leishmaniasis (CL), mucocutaneous leishmaniasis (MCL), and visceral leishmaniasis (VL) have a high impact in several countries of the world due to their morbidity and mortality rates. Many *Leishmania* species act as etiological agents of cutaneous and MCL; one of the most well known agents of the diffuse cutaneous form is *Leishmania amazonensis* and MLC forms is *L. brasiliensis*. On the other hand, *L. donovani* and *L. chagasi* are the only etiological agents of VL. The CL is characterized by lesions on the face, hands, and/or feet. MCL involves the nasal, oral, and pharyngeal mucosa and causes difficulty in eating as well as an increased risk of secondary infections that have a significant mortality rate (Murray et al., 2005; Piscopo and Mallia, 2007; David and Craft, 2009). The VL is characterized by fever, weakness, and fatigue are worsened by anemia, which is caused by a persistent inflammatory state, hypersplenism, and sometimes by bleeding. As the disease advances, splenomegaly and hepatomegaly can increase, causing abdominal distension and pain. These VL symptoms often persist for a long time before the patient either seeks medical care or dies from secondary infections, severe anemia, or organ failure (Chappuis et al., 2007; Piscopo and Mallia, 2007).

The life cycle of *Trypanosoma cruzi*, the etiological agent of Chagas disease, involves blood sucking by triatomine bugs that serve as insect vectors and a mammalian host where the parasite undergoes an obligate intracellular amastigote replicative form and an extracellular non-replicative trypomastigote form in the bloodstream. The epimastigote replicative form and a non-replicative infective metacyclic trypomastigote form are also observed in the insect vector. This disease is mainly transmitted by insect vectors feces, but transmission may also occur by blood transfusion, organ transplant, congenital and oral routes, and laboratory accidents. The prevalence and incidence of Chagas disease and the associated mortality are constantly changing as a consequence of vector control programs, rural-urban human migration, and changes in the socio-economic status of risk areas (Lewinsohn, 2003; Moncayo and Ortiz Yanine, 2006).

Despite the fact that most people infected with the parasite never appear to become symptomatic, Chagas disease presents in two stages: the acute stage, which appears shortly after the infection, and the chronic stage, which may last several years. In the acute phase, fever, lymphadenopathies, hepatomegaly, and splenomegaly may appear, as well as more severe problems, such as acute myocarditis and meningoencephalitis, which may be fatal in 2–8% of the cases in the absence of specific treatment (Moncayo and Ortiz Yanine, 2006; Develoux et al., 2009).

African trypanosomiasis or sleeping sickness occurs only in 36 countries in sub-Saharan Africa that contain vector insects of the genus *Glossina* (tsetse flies). Sleeping sickness is a serious public health problem in these countries because 55 million people are at risk of infection and there are an estimated 30,000 new cases annually. If left untreated, the disease is almost always fatal. Its etiologic agents are two subspecies of *Trypanosoma brucei*, *T. b. gambiense* in West Africa, and *T. b. rhodesiense* in East Africa, and the last is responsible for most severe form of the same disease. The trypanosomes of the *brucei* group primarily infect connective tissues. Hemolytic anemia occurs in the early stages of infection, followed by lymph node, spleen, and liver hypertrophy and cardiovascular and endocrine disorders. In the final phase of the infection, the parasites reach the nervous system and cause inflammatory reactions that lead to the meningoencephalitis associated with the clinical aspects of sleeping sickness (Gehrig and Efferth, 2008; Astelbauer and Walochnik, 2011).

## Current Chemotherapy Against Leishmaniasis, Chagas Disease, and Sleeping Sickness and Impact of Parasite Resistance

Chemotherapy is the main tool used to control parasitic infections. The drugs available for these parasites control are often ineffective and sometimes life-threatening, adverse side effects, and some of these drugs require hospitalization (Croft et al., 2006; Ashutosh and Goyal, 2007; Piscopo and Mallia, 2007).

The drugs available for leishmaniasis infections are the pentavalent antimonial formulations of sodium stibogluconate (Pentostan) and *N*-methyl glucantime (Glucantime), which target the amastigotes of both CL and VL. Of the various proposed mechanisms of action for these two drugs, we can mention the production of ATP inhibition of the citric acid cycle and glycolysis by converting the pentavalent antimony the active trivalent antimony which is more active and more toxic, inhibition of oxidation of fatty acids in amastigotes and induction of apoptosis and inhibition of the enzyme DNA topoisomerase (Berman et al., 1987; Frézard et al., 2009). Variations in the clinical responses to these drugs have been a persistent problem in the treatment of leishmaniasis over the past 50 years due to intrinsic differences in species sensitivity and the development of resistance. When these drugs are ineffective or cannot be prescribed, treatment with amphotericin B, pentamidine, or paromomycin is indicated. However, as none of these drugs are free of adverse effects, the search for alternative therapeutic agents is essential. The combination of amphotericin B liposome is an alternative employed to reduce adverse effects, increasing the efficiency of the drug. As fluconazole and ketoconazole, azoles which initially were designed for the treatment of fungal infections, have been used for treating CL. Miltefosine has been recently approved as the first oral drug for VL. It yields cure rates of approximately 98% and is used to treat cases of resistance to antimoniates; however, it has been shown to induce parasite resistance *in vitro* (Croft et al., 2006; Santos et al., 2008; Goto and Lindoso, 2010; Tiuman et al., 2011).

Nifurtimox (Nif; Bayer 2502) and benznidazole (Bz; RO 7-1051) have been used for the treatment of Chagas disease since the end of 1960. The mechanism of action of Nif is based on a partial deficiency in the ability of *T. cruzi* to detoxify free radicals. Nif leads to the formation of nitro anion radicals that in turn produce highly toxic reduced oxygen metabolites. However, Nif is no longer commercially available. Instead of producing oxidative damage, the Bz mechanism of action might involve covalent modifications of parasite proteins, lipids, and DNA by nitro reduction intermediates (Coura and de Castro, 2002). The likelihood of curing Chagas disease with Nif and Bz varies according to the phase of the disease, the period of treatment, the dosage, and the age of the patient. Usually, satisfactory results are achieved when the patients are treated in the acute phase, in recent chronic infection, in congenital infection, and after laboratory accidents. The major limitation of these compounds is the low efficacy in the treatment of patients in the chronic phase of the disease (Coura and de Castro, 2002).

Treating infections with Nif may lead to collateral effects such as psychic alterations, anorexia, excitability, sleepiness, and digestive manifestations (nausea, vomiting, and diarrhea). Doses of Bz for Chagas disease treatment may cause hypersensitivity leading to dermatitis with cutaneous eruptions and generate neuropathologies such as paresthesia and polyneuritis of the peripheral nerves. However, the most serious reaction to Bz is the depression of bone marrow that leads to thrombocytopenic purpura and agranulocytosis. Due to these characteristic side effects, Nif and Bz should not be used by elderly or pregnant patients or by patients presenting any severe disease associated with Chagas disease, such as cardiac, respiratory, renal, or hepatic insufficiency, systemic infection, and neoplasia (Coura and de Castro, 2002).

The use of nanotechnology as liposomes, antibody conjugates, and nanoparticles as drug carriers can overcome anatomical barriers and take the drug directly to the site of action, reaching the target microorganisms, and reducing their side effects. Alternatively, amphotericin B can be used as a second line treatment in Chagas disease using nano-drug delivery systems (Yardely and Croft, 1999; Romero and Morilla, 2010). The E1224 azole compound, a prodrug transformed into ravuconazole *in vivo*, discovered and developed by Esai Pharmaceuticals had preclinical and clinical phase I studies completed. This promising compound for the treatment of Chagas disease, has been tested in phase II clinical studies in adult patients in Bolivia [Drugs for Neglected Diseases Iniciative (DNDi), 2012]. Furthermore, the ravuconazol was evaluated extensively in animal models (Diniz et al., 2010).

The treatment for sleeping sickness depends on the stage of the disease. The drugs used in the first stage of the disease have a low toxicity and are easy to administer. Therefore, early identification results in an increased potential for curing the disease. Treatment success in the second stage of disease depends on a drug that can cross the blood-brain barrier to reach the parasite. Such drugs are toxic and complicated to administer. There are four drugs typically used for the treatment of sleeping sickness. Early infections are treated with pentamidine and suramin. Pentamidine is used for the treatment of the first stage of *T. b. gambiense* sleeping sickness. It has few undesirable effects and is generally well tolerated by patients. Suramin is used for the treatment of the first stage of sleeping sickness by *T. b. rhodesiense* but can cause allergic reactions and some undesirable effects in the urinary tract. In their final stages, both forms of infections are treated with melarsoprol, a drug derived from arsenic. This drug has many side effects, the most dramatic of which is reactive encephalopathy, which can be fatal, and kills up to 5% of patients. It is supposed that the mechanism of action is the interaction of the drug with a transporter specific for adenosine reducing the absorption of these nucleoside by the parasite and by the arsenic binding to glycerol 3-phosphate dehydrogenase, resulting in inhibition of glycolysis, and low levels of ATP (Denise and Barret, 2001). Another option is eflornithine, which is less toxic but only effective against *T. b. gambiense* infections. A combination treatment with nifurtimox and eflornithine was recently (2009) introduced, but is not effective for *T. b. rhodesiense* (Wilkinson and Kelly, 2009; Astelbauer and Walochnik, 2011; Jacobs et al., 2011).

One major drawback to the treatment of leishmaniasis, Chagas disease, and sleeping sickness is the emergence of resistance to current chemotherapeutics. Due to their high toxicity, drugs usually used for the treatment of these diseases have to be administered in low doses, allowing drug resistance to develop. Because there are few drugs available for the treatment of these parasitoses, resistance has a considerable impact on the control of these maladies.

The primary mechanism generally observed in parasite resistance is a decrease in the drug concentration within the parasite cell. The drug level may be lowered by a variety of mechanisms, including decreased uptake, increased efflux, and inhibition of drug activation and inactivation of active drug by the metabolism. In recent years, a large-scale increase in clinical resistance of *Leishmania* to pentavalent antimonials has been reported. The mechanisms of resistance of *Leishmania* spp. and *T. cruzi* against the chemotherapeutics developed in the field are not elucidated, and most of our knowledge stems from work on laboratory mutants (Nogueira et al., 2006; Ashutosh and Goyal, 2007).

The resistance mechanisms in *T. b. rhodesiense* and *T. b. gambiense*, recently reviewed by Gehrig and Efferth (2008), are frequently mediated by a reduced net drug uptake. Another drug resistance mechanism for melarsoprol is the overexpression of efflux pumps (Mäser et al., 2003).

A study using RNA interference in *T. brucei* showed the loss of function of a nitroreductase and an amino acid transporter necessary for activation of the prodrug nifurtimox and acquiring of eflornithine, respectively, showing the mechanisms of resistance to these two drugs (Baker et al., 2011).

## Plants as Promising Sources of Anti-Leishmanial and Anti-Trypanosomal Compounds

Considering the lack of vaccines, the toxicity of the chemotherapies, the side effects of the treatment, and the resistance of the parasites to the drugs, there is an evident need to discover drugs that can be used as therapeutics for leishmaniasis, sleeping sickness, and Chagas disease. In recent years, many pharmacologically active and microbicidal compounds derived from plants have been discovered (Cowan, 1999; Alviano and Alviano, 2009; Izumi et al., 2011), indicating that phytoscience is important in the search of novel compounds with a potential to control these diseases.

In fact, promising results have been obtained by our group and others in the search of plant-derived crude extracts, essential oils, and compounds with activity against pathogenic microorganisms, including *Leishmania* spp., *T. cruzi* (Alviano and Alviano, 2009; Izumi et al., 2011), and *T. brucei* (Gehrig and Efferth, 2008). Useful antiprotozoal phytocompounds can be divided into several categories summarized in Table 1.

**Table 1:** **Plants and identified antiprotozoal bioactive phytocompounds**.

Scientific name	Major components	Effects	Parasites	Reference
*Drymis brasiliensis*	Polygodial	Cell proliferation (promastigotes), Cell viability (trypomastigotes), mitochondrial changes, nuclear changes, plasma membrane damages (promastigotes)	*L. amazonensis*, *L. braziliensis*, *L. chagasi*, *L. major*, *Trypanosoma cruzi*	Corrêa et al. (2011)
*Baccharis retusa*	Sakuranetin	Cell proliferation (promastigotes), Cell viability (trypomastigotes), activity in intracellular amastigotes	*L. amazonensis*, *L. braziliensis*, *L*. *chagasi*, *L. major*, *T. cruzi*	Grecco et al. (2012)
*Ocimum gratissimum*	Eugenol	Cell lysis e proliferation (promastigotes), mitochondrial changes, stimulation of NO production in macrophages	*L. amazonensis Trypanosoma brucei rhodesiense*	Ueda-Nakamura et al. (2006), Abiodun et al. (2012)
*Croton cajucara*	Linalool, acetyl aleuritolic acid	Mitochondrial changes (promastigotes), activity in intracellular amastigotes. Cell viability (trypomastigotes), activity in intracellular amastigotes, mitochondrial, and kinetoplast changes (epimastigotes)	*L. amazonensis, T. cruzi*	Rosa et al. (2003), Campos et al. (2010)
*Piper claussenianum*	Trans-nerolidol	Cell proliferation, activity in intracellular amastigotes, increased NO production in infection macrophages, decreased arginase activity of the parasite	*L. amazonensis*	Marques et al. (2010, 2011)
*Lippia alba*	Geranial, neral, geraniol, and trans-β-caryophyllene	Cell proliferation (epimastigotes) and intracellular amastigotes activity	*T. cruzi*	Escobar et al. (2010)
*Lippia origanoides*	Oxygenated monoterpenes	Cell proliferation (promastigotes)	*L. chagasi*	Escobar et al. (2010)
*Moringa stenopetala*	Benzyl-isothiocyanate	Cell proliferation (bloodstream forms)	*Trypanosoma brucei brucei*	Nibret and Wink (2010)
*Hagen abyssinica*	Ledol	Cell proliferation (bloodstream forms)	*T. b. brucei*	Nibret and Wink (2010)
*Leonotis ocymifolia*	Caryophyllene oxide	Cell proliferation (bloodstream forms)	*T. b. brucei*	Nibret and Wink (2010)
*Cupania cinerea*	Cupacinoside, taraxerol	Cell proliferation (bloodstream forms)	*T. b. rhodesiense*	Gachet et al. (2011)
*Kola acuminata*	Proanthocyanidin	Cell proliferation (bloodstream forms and procyclic forms), cell lysis and changes in plasma membrane	*T. brucei* (clone221a)	Kubata et al. (2005)
*Salvia hydrangea*	Salvadione, perovskone	Cell proliferation (bloodstream forms)	*T. b. rhodesiense*	Farimani et al. (2011)
*Carlina acaulis*	*Carlina* oxide (polyacetylene)	Cell proliferation (bloodstream forms)	*T. b. brucei*	Herrmann et al. (2011)
*Syzygium aromaticum*	Eugenol	Cell proliferation and viability (epimastigotes and bloodstreams trypomastigotes) and loss of nuclear content, and masses of condensed chromatin (trypomastigotes)	*T. cruzi*	Santoro et al. (2007)
*Ocimum basilicum*	Linalool	Cell proliferation and viability (epimastigotes and bloodstreams trypomastigotes), cytoplasmic extraction and nuclear alteration (epimastigotes)	*T. cruzi*	Santoro et al. (2007)
*Achillea millefolium*	Chamazulene	Cell proliferation and viability (epimastigotes and bloodstream trypomastigotes)	*T. cruzi*	Santoro et al. (2007)
*Zanthoxylum chiloperone*	Chantin-6-one	Cell lysis (bloodstream trypomastigotes), anti amastigotes activity and *in vivo* activity in infected mice	*T. cruzi*	Ferreira et al. (2011)
*Centaurea salmantica*	Cynaropicrin	Cell proliferation (bloodstream trypomastigotes), *In vivo* activity (reduction of parasitemia)	*T. b. rhodesiense, T. b. gambiense*	Zimmermman et al. (2012)
*Lippia sidoides*	Thymol	Cell proliferation, accumulation of lipid droplets, wrinkled, or ruptured membranes and the loss of cytoplasm (promastigotes)	*L. amazonensis*	Medeiros et al. (2011)
*Cymbopogon citratus*	Citral	Cell proliferation, ultrastructural alterations like mitochondrial and kinetoplast swelling and disruption of nuclear membrane, loss of mitochondrial membrane potential and other alterations	*L. infantum, L. major, L. tropica*	Machado et al. (2012)
*Ambrosia scabra*	Psilostachyin C	Cell proliferation (epimastigotes and promastigotes), ultrastructural changes, anti amastigotes activity, *In vivo* activity (reduction of parasitemia)	*T. cruzi, L. amazonensis, L. mexicana*	Sülsen et al. (2011)
*Chamomilla recutita*	(−) α-bisabolol	Cell proliferation (promastigotes)	*L. infantum*	Morales-Yuste et al. (2010)
*Xanthium strumarium*	Xanthatin	Cell proliferation (bloodstream forms), mitochondrial membrane potential reduction, trypanothione reductase inhibition	*T. b. brucei*	Nibret et al. (2011)
*Saussurea costus*	Sesquiterpene lactones	Cell proliferation (bloodstream forms)	*T. b. rhodesiense*	Julianti et al. (2011)
*Piper aduncum*	2′,6′-dihydroxy-4′-methoxychalcone	Cell proliferation (promastigotes), mitochondrial damage, anti-intracellular amastigote activity	*L. amazonensis*	Torres-Santos et al. (1999)
*Piper rusbyi*	Kavapyrone, Flavokavain	Cell proliferation (promastigotes) *In vivo* activity (lesion size reduction)	*L. amazonensis, L. braziliensis, L. donovani*	Flores et al. (2007)
*Piper auritum*	Safrole	Cell proliferation (promastigotes), anti-intracellular amastigote activity	*L. major, L. mexicana, L. braziliensis, L. donovani*	Monzote et al. (2010)
*Piper regnellii*	Eupomatenoid-5	Cell proliferation (promastigotes and axenic amastigotes), anti-intracellular amastigotes Ultrastructural alteration and lipoperoxidation in the cell membrane (epimastigotes and bloodstream forms)	*L. amazonensis Trypanosoma cruzi*	Vendrametto et al. (2010), Pelizzaro-Rocha et al. (2011)
*Aframomum sceptrum*	Sceptrumlabdalactone B	Cell proliferation (promastigotes and bloodstream forms)	*L. donovani T. b. brucei*	Cheikh-Ali et al. (2011)

## Anti-Leishmanial Compounds

Essential oils are known to possess a wide variety of hydrophobic compounds with antimicrobial potential. The ability to diffuse across cell membranes certainly gives to those molecules some advantage in targeting cellular components, being a valuable research option for the search of bioactive compounds (Bakkali et al., 2008). The *Ocimum gratissimum* essential oil and eugenol, its major component, was tested on the growth, viability, and ultrastructural alterations of the amastigote and promastigote forms of *L. amazonensis*, as well as on the interaction of these flagellates with mouse peritoneal macrophages, concomitant with nitric oxide production stimulation by the infected macrophages. Significant mitochondrial alterations occurred at the ultrastructural level of the parasite, such as remarkable swelling, disorganization of the inner membrane, and an increase in the number of cristae after treatment of parasites with *O. gratissimum* essential oil. However, mouse macrophages were unaffected under the same conditions. In addition, nitric oxide production was dramatically stimulated when mouse peritoneal macrophages were treated with 150 μg/ml essential oil, both before and after infection with *L. amazonensis*. Concomitantly, the association indexes were drastically lower in the latter conditions, compared to the control system (Ueda-Nakamura et al., 2006).

**Figure 1:**
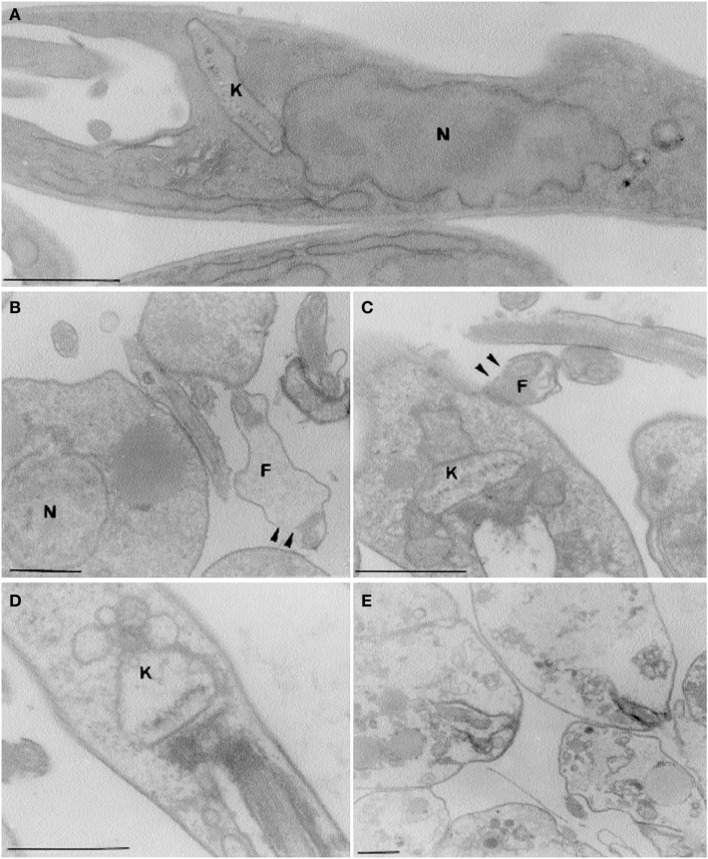
**Effects of linalool-rich essential oil (15.0 ng/ml) extracted from *C. cajucara* on the promastigote stage of *L. amazonensis*, as observed by transmission electron microscopy**. **(A)** Control parasites; **(B–E)** parasites treated for 5 **(B)**, 10 **(C)**, 15 **(D)**, and 30 **(E)** min, showing promastigotes with different degrees of damage. Note the disruption of the flagellar membranes [arrowheads in **(B,C)**], the mitochondrial swelling **(C,D)**, and the gross alterations in the organization of the nuclear and kinetoplast chromatins **(C,D)**. In the presence of the essential oil, the parasites were completely destroyed after 30 min of treatment **(E)**. N, nucleus; K, kinetoplast, F, flagellum. Bars, 1 μm. Image reproduced with permission from © Rosa et al. (2003) American Society for Microbiology.

The linalool-rich essential oil extracted from the leaves of *Croton cajucara*, has effects on *L. amazonensis* parasites, on the interaction of these flagellates with mouse peritoneal macrophages and on nitric oxide production by the infected macrophages. The median lethal doses and absolute lethal doses of the essential oil and linalool-rich essential oil from *C. cajucara* for promastigotes and amastigotes were very low. Mitochondrial swelling and alterations in the organization of the nuclear and kinetoplast chromatins were observed by electron microscopy when *L. amazonensis* parasites were treated with the essential oil from *C. cajucara* (Figure 1). The viability of mouse macrophages was unaffected by the same concentrations. When the macrophages were pre-treated with *C. cajucara* essential oil, as well as when the macrophages were pre-infected with the parasites and then treated with the essential oil, the association indexes were 50% lower than those for the control system. In addition, the macrophages pre-treated with *C. cajucara* essential oil produced twice the amount of nitric oxide as the untreated macrophages (Rosa et al., 2003).

Piper species have been reported to have activity against *Leishmania* parasites (Torres-Santos et al., 1999; Flores et al., 2007; Sarkar et al., 2008). Our group recently showed that the leaf essential oil from *Piper claussenianum* was able to inhibit the growth of *L. amazonensis* promastigotes with an IC_50_ of 0.0038% (Marques et al., 2010). Trans-nerolidol is a major component of this essential oil. We also examined the effect of the essential oil on the interaction between parasites and host cells and its activity against intracellular amastigotes. The IC_50_ concentration used against promastigotes yielded a 31% reduction in the percentage of infected macrophages and led to a 17% increase in the production of NO by macrophages. The activity of the enzyme arginase may be able to modulate nitric oxide production by macrophages by inhibiting nitric oxide synthase (iNOS). Promastigotes were grown in the presence of the IC_50_ of the essential oil and showed a 62% reduction in arginase activity. (Marques et al., 2011). Monzote et al. (2010) described the anti-leishmanial activity of the essential oil from *Piper auritum*. In that study, essential oil inhibited the growth of promastigotes in all species of *Leishmania* used, with IC_50_ values between 12.8 and 63.3 μg/mL. In addition, piper-oil inhibited the growth of intracellular amastigotes of *L. donovani* at non-toxic concentrations (Monzote et al., 2010).

## Anti-Trypanosomial Compounds

**Figure 2:**
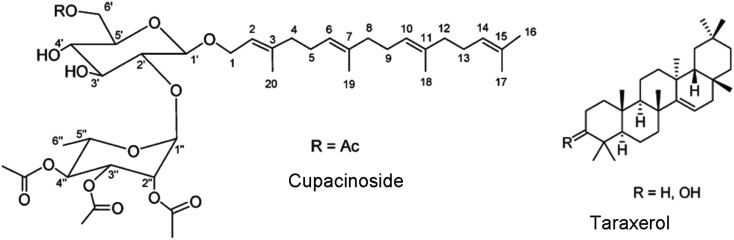
**Structure of cupacinoside and taraxerol**. Reprinted (adapted) with permission from © Gachet et al. (2011) American Chemical Society.

One new diterpene glycoside, cupacinoside, and one known compound, taraxerol (Figure 2) from *n*-hexane, and dichloromethane extracts from the bark of *Cupania cinerea* (Sapindaceae) have shown significant *in vitro* activity against one of the etiologic agents of sleeping sickness. Cupacinoside and taraxerol showed IC_50_ values <10 μM against *T. b. rhodesiense*, with taraxerol exhibiting only low cytotoxicity against rat skeletal myoblast cell line (L-6 cells; Gachet et al., 2011).

**Figure 3:**
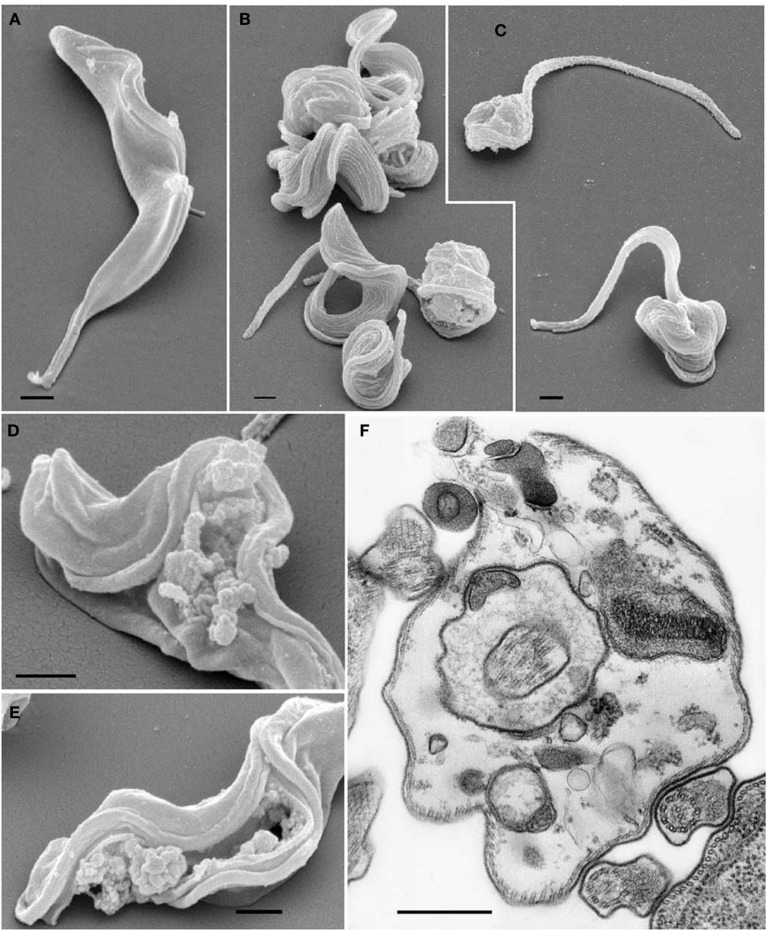
**Effects of *Kola acuminate* proanthocyanidin (50 μg) on bloodstream form trypanosomes observed by scanning and transmission electron microscopy**. **(A)** Control parasites showing the normal cell morphology and membrane integrity. **(B,C)** Parasites treated for 6 h showing morphological changes and the rounding up of the cells. **(D,E)** Cells showing disintegrated cell membranes and loss of cytoplasmic contents caused by the effect of the drug. **(F)** TEM of a trypanosome cell confirming the necrotic process of cell membrane disintegration and loss of cytoplasmic contents that led to cell death. Scale bar: 1 μm. Image reproduced with permission from © Kubata et al. (2005) Elsevier.

A proanthocyanidin isolated from *Kola acuminate* seeds was able to inhibit the proliferation and cause the lysis of *Trypanosoma brucei* bloodstream forms *in vitro*. The *in vivo* effect was trypanostatic and prolonged the survival of infected animals that were treated with this substance. Additionally, it was not toxic to the human epidermoid carcinoma cells (KB 3-1), but it caused ultrastructural changes in parasites, such as rupture of the plasma membrane and vesicles and the formation of multivesicular bodies in lysosome-like organelles, when the parasites were treated for 6 h with the compound (Figure 3; Kubata et al., 2005).

**Figure 4:**
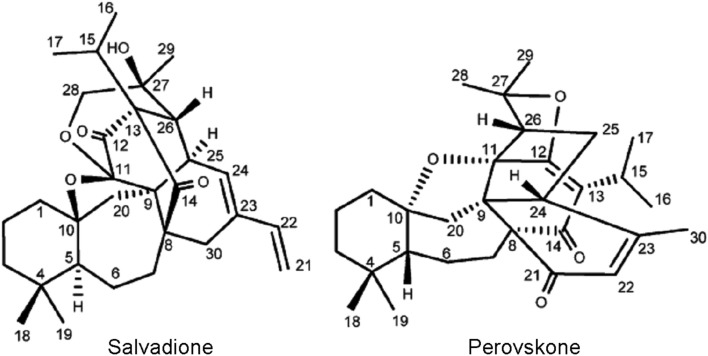
**Structure of salvadione and perovskone**. Reprinted (adapted) with permission from © Farimani et al. (2011) American Chemical Society.

Salvadione and perovskone (Figure 4), two new triterpenoids with rare carbon skeletons isolated from aerial parts and flowers of *Salvia hydrangea* were used in the treatment of leishmaniasis in Iran. These compounds were tested *in vitro* against *T. b. rhodesiense* and exhibited moderated potency with IC_50_ values 4.33 and 15.92 μM, respectively and good selectivity index for L-6 cells (Farimani et al., 2011).

A study using essential oils of cloves (*Syzygium aromaticum*), basil (*Ocimum basilicum*), and a yarrow (*Achillea millefolium*) and the main constituents, eugenol and linalool showed activity on *T. cruzi* bloodstream trypomastigotes and epimastigotes forms. The essential oils inhibited epimastigotes proliferation, caused trypomastigotes lysis and ultrastructural changes in both forms, mainly in the nucleus. In epimastigotes forms was observed shrinkage of the nuclear material with separation of the nuclear membrane and in trypomastigotes forms loss nuclear material and masses of condensed chromatin appeared (Santoro et al., 2007).

Acetyl aleuritolic acid, a terpene isolated from the methanolic extract of stem bark of *C. cajucara* showed significant trypanocidal effect in trypomastigotes of a strain isolated from wild reservoirs (GLT291), genotype TCI as clone Dm28c. was also effective against intracellular amastigotes and did not show a significant effect on proliferative epimastigotes. This compound was also able to inhibit the activity of trypanothione reductase, an important enzyme in the regulation of redox balance and defense against oxidative stress in this parasite (Campos et al., 2010). Xanthatin, a sesquiterpene lactone, was described by Nibret et al. (2011) as strong trypanocidal agent with an IC_50_ value of 2.63 μg/mL. According to the authors, it seems that the biological activity of the compound is a result of its effect in inducement of apoptosis in trypanosomes as evidenced by a reduction in mitochondrial membrane potential. Furthermore, xanthatin was able to inhibit the two key enzymes involved in the inflammatory process, cyclooxygenase and 5-lipoxygenase, which can be very interesting in diseases that cause this kind of response (Nibret et al., 2011).

## Conclusion

At a time when there is an urgent need for the efficient treatment of leishmaniasis, Chagas disease, and sleeping sickness, this review shows that plant-derived extracts and compounds exhibit significant antiprotozoal activities *in vitro* and emphasizes the importance of phytoscience in the search for novel anti-leishmanial and anti-trypanosomal therapeutic agents.

## Conflict of Interest Statement

The authors declare that the research was conducted in the absence of any commercial or financial relationships that could be construed as a potential conflict of interest.
